# Exploring the practice, confidence and educational needs of hospital pharmacists in reviewing antimicrobial prescribing: a cross-sectional, nationwide survey

**DOI:** 10.1186/s12909-021-02664-1

**Published:** 2021-04-23

**Authors:** Sharmila Khumra, Andrew A. Mahony, Phillip J. Bergen, Amy T. Page, Rohan A. Elliott

**Affiliations:** 1 Centre for Medicines Use and Safety, Faculty of Pharmacy and Pharmaceutical sciences, Monash University, 381 Royal Parade, Parkville, Victoria 3052 Australia; 2 Department of Pharmacy, Austin Health, 145 Studley Road, Heidelberg, Victoria 3084 Australia; 3 Department of Infectious Diseases, Austin Health, 145 Studley Road, Heidelberg, Victoria 3084 Australia; 4 Department of Medicine, University of Melbourne, Melbourne, Victoria 3010 Australia; 5 Department of Pharmacy, Alfred Health, 55 Commercial Rd, Melbourne, Victoria 3004 Australia

**Keywords:** Australia, Antimicrobial stewardship, Pharmacists, Education, Knowledge, Survey

## Abstract

**Background:**

Antimicrobial stewardship (AMS) programs are usually limited in resources and scope. Therefore, wider engagement of hospital pharmacists in reviewing antimicrobial orders is necessary to ensure appropriate prescribing. We assessed hospital pharmacists’ self-reported practice and confidence in reviewing antimicrobial prescribing, and their knowledge in making AMS interventions.

**Methods:**

We conducted an Australia-wide, cross-sectional survey in October 2017. A link to the online survey was emailed to hospital pharmacists via the Society of Hospital Pharmacists of Australia. Factors associated with higher knowledge scores were explored using linear regression models.

**Results:**

There were 439 respondents, of whom 272 (61.7%) were from metropolitan public hospitals. Pharmacists were more likely to assess the appropriateness of intravenous, broad-spectrum or restricted antibiotics than narrow-spectrum, oral antibiotics within 24–72 h of prescription; *p* < 0.001. Fifty percent or fewer respondents were confident in identifying AMS interventions related to dose optimization based on infection-specific factors, bug-drug mismatch, and inappropriate lack of spectra of antimicrobial activity. The median knowledge score (correct answers to knowledge questions) was 6 out of 9 (interquartile range, 5–7); key gaps were noted in antimicrobials’ anaerobic spectrum, beta-lactam allergy assessment and dosing in immunocompromised patients. Clinical practice in inpatient areas, registration for 3–5 years and receipt of recent AMS education were associated with higher knowledge scores. More interactive modes of education delivery were preferred over didactic modes; *p* ≤ 0.01.

**Conclusion:**

Gaps in practice, confidence and knowledge among hospital pharmacists were identified that could inform the design of educational strategies to help improve antimicrobial prescribing in Australian hospitals.

**Supplementary Information:**

The online version contains supplementary material available at 10.1186/s12909-021-02664-1.

## Background

Inappropriate prescribing of antimicrobials in hospitalised patients can cause harm through the selection of antimicrobial-resistant pathogens, drug toxicity, allergic reactions and opportunistic infections (e.g. *Clostridioides difficile* and fungal infections) [1–3]. Antimicrobial stewardship (AMS) is an evidence-based strategy that promotes safe and appropriate prescribing of antimicrobials to improve patient safety and clinical and economic outcomes [4]. Hospital-wide AMS programs are usually coordinated by a multidisciplinary team including a pharmacist knowledgeable in infectious disease (ID) pharmacotherapy (ID/AMS pharmacist). A core activity of the AMS team is reviewing the appropriateness of antimicrobial orders, with intervention and direct feedback to prescribers to optimize prescribing (commonly referred to as prospective audit and feedback [PAF]) [5]. Common AMS interventions identified through review of antimicrobial prescribing include narrowing the spectrum of antimicrobial activity based on microbiology tests, discontinuing antimicrobials when no longer needed, intravenous-to-oral antimicrobial switch, and dose optimization [6]. However, AMS teams are generally limited in their scope and capacity to oversee prescribing of all antimicrobials for every inpatient. Given the labour- and resource-intensive nature of PAF, the focus of this activity is often on specific antimicrobials (e.g. broad-spectrum agents) or infections, or wards with a high burden of antimicrobial prescribing such as intensive care or haematology units [5, 6]. Therefore, wider engagement of frontline healthcare providers not directly involved in AMS programs, including hospital pharmacists, is necessary to ensure optimal use of antimicrobials, especially those agents not routinely reviewed by AMS teams [7].

Hospital pharmacists could be drivers of sustainable, wide-reaching PAF. However, little is known about their confidence or knowledge in making AMS interventions. Previously published studies assessed self-reported practice, confidence and knowledge around antimicrobial use, resistance and stewardship, but mostly focused on medical [8–11] or pharmacy students [12, 13], and hospital doctors [14–16] or nurses [17]. These studies revealed low to medium mean knowledge scores among participants (34–69%) [8, 11, 12, 17]. One study surveyed Australian and French hospital pharmacists with an interest or involvement in AMS programs [18]. Both groups indicated a lack of confidence in adjusting the dose and frequency of antimicrobials based on pharmacodynamic parameters and participating in AMS team care rounds. Knowledge in making AMS interventions upon review of antimicrobial prescribing was not assessed.

The primary aim of this study was to assess hospital pharmacists’ self-reported practice and confidence in reviewing antimicrobial prescribing, and to assess their knowledge in making AMS interventions. Such information will help inform the development of education and training programs for hospital pharmacists to embed AMS principles in their practice. Secondary aims were to identify factors associated with hospital pharmacists’ knowledge scores and level of confidence, and to identify their preferred modes of AMS education delivery.

## Methods

### Study design and population

We conducted a cross-sectional, nationwide, online survey of registered and intern (pre-registration) pharmacists employed in Australian hospitals.

### Survey tool and administration

A 38-item survey (Additional file 1) was designed by two ID pharmacists and one ID physician, to evaluate respondents’: (i) demographics (multiple-choice questions [MCQ]); (ii) self-reported practice of reviewing appropriateness of antimicrobial orders after the initial prescription (MCQ with optional free-text comments box); (iii) self-reported confidence in identifying AMS interventions during antimicrobial review (Likert-scale answers); (iv) knowledge of AMS interventions (clinical vignettes with MCQ, including three vignettes with MCQ adapted from previously published tools [8, 14, 15]), and (iv) perceived usefulness of different modes of AMS education delivery (Likert-scale answers). In section ii, review of appropriateness was defined as reviewing the choice of antimicrobial agent, dosage, route and duration. It was assumed that all antimicrobial orders were reviewed by a pharmacist at the time of initial prescription, so survey questions focused on when pharmacists would re-evaluate ongoing appropriateness, for example, based on further clinical or microbiology data.

Face validity of the survey was performed by having 11 registered pharmacists, one intern pharmacist and two ID physicians complete the survey and provide feedback on readability, relevance of questions and length which enabled fine tuning.

Registered and intern pharmacists who were members of the Society of Hospital Pharmacists of Australia (SHPA) (*n* = 3472) were sent an invitation to complete the survey (including survey link) via the SHPA weekly email newsletter. The link was re-sent via the newsletter one and 3 weeks later. It was administered online using SurveyMonkey (SurveyMonkey Inc. San Mateo, California, USA, www.surveymonkey.com). The survey was available for 6 weeks from 18 October to 29 November 2017; no incentive was offered to participants. The survey was voluntary and anonymous, and answering each question was optional.

### Data analysis

Data were analysed using Stata software (StataCorp. 2019. Stata Statistical Software: Release 16. College Station, TX). Descriptive analysis was performed using the total number of responses for each question as the denominator for that question. Categorical data were compared using the chi-square test. Responses to questions about confidence in identifying AMS interventions were collapsed from a 5-point Likert scale into two categories: *confident* (‘very confident’ or ‘confident’) and *lacking confidence* (‘somewhat confident’, ‘not confident at all’, or ‘I don’t know how I feel’). Answers to the knowledge test were also collapsed into two categories: *correct* (the correct answer was provided) and *incorrect* (an incorrect answer was provided or ‘I’m not sure. I would have to look it up’). For respondents who answered all nine knowledge-based questions, an overall knowledge score was calculated as the total sum of correct answers, to determine the median knowledge score and interquartile range (IQR). Associations between respondents’ demographics and their knowledge scores were evaluated by univariate and multivariate analyses using linear regression. A *p*-value < 0.05 was deemed statistically significant, and 95% confidence intervals are presented where appropriate. Responses regarding the usefulness of different modes of education delivery were collapsed into three categories: ‘very useful/useful’, ‘not useful/not useful at all’ and ‘neutral’. Responses to open-ended questions were reviewed for key terms or concepts. For simplicity, intern and registered pharmacists practising in hospitals are hereafter collectively referred to as ‘hospital pharmacists’.

## Results

Of the 465 survey respondents (response rate 13.4%), 26 were excluded: 2 did not work in a hospital and 24 only completed the demographics questions. Data from the remaining 439 respondents who had either completed (221/439, 50.3%) or partially completed (218/439, 49.7%) the survey were analysed. Respondent demographics are shown in Table 1.

**Table 1:** Demographics of survey respondents

	N (%)
*State or Territory (n = 439)*	
Victoria	164 (37.4)
Queensland	82 (18.7)
New South Wales	56 (12.7)
Western Australia	47 (10.7)
South Australia	33 (7.5)
Tasmania	28 (6.3)
Northern Territory	19 (4.3)
Australian Capital Territory	10 (2.3)
* Health Service (n = 439)*	
Major city, public	272 (61.7)
Regional, public	108 (24.6)
Major city, private	36 (8.2)
Remote or very remote public	13 (2.9)
Regional, private	10 (2.3)
* The hospital has an individual or team dedicated to the review of antimicrobials* (*n* = 439)	
Yes	355 (80.9)
No	73 (16.6)
I don’t know	11 (2.5)
* Years of pharmacy registration (n = 434)*	
> 10 years	182 (41.9)
6 to 10 years	113 (26.0)
3 to 5 years	66 (15.2)
< = 2 years	53 (12.2)
Intern/pre-registration	20 (4.6)
* Highest level of pharmacy education (n = 439)*	
Bachelor of Pharmacy	181 (41.2)
Postgraduate diploma	80 (18.2)
Postgraduate certificate	79 (18.0)
Postgraduate Master degree	77 (17.5)
Preregistration Master degree	11 (2.5)
PhD	8 (1.8)
Other (e.g. Honours)	3 (0.7)
* Current main area of hospital practice (n = 439)*	
Working in ward/inpatient area of high antimicrobial use (e.g. intensive care unit)	79 (18.0)
Any other ward/inpatient area	206 (46.9)
Antimicrobial stewardship/infectious diseases service	32 (7.3)
Medication safety/Quality use of medicines service	29 (6.6)
Outpatient dispensary	26 (5.9)
Non inpatient areas (e.g. day oncology, hospital in the home [HITH])	17 (3.9)
Other (e.g. manufacturing, administration, information technology)	50 (11.4)
* Type of antimicrobial stewardship (AMS) education undertaken/received in the past 12 months (n = 439)*^a^	
Informal education in the workplace	246 (56.0)
Self-directed learning	208 (47.4)
Workshop or seminar	115 (26.2)
None	94 (21.4)
Web-based module/course	94 (21.4)
Lecture	92 (21.0)
Postgraduate studies (university)	53 (12.0)

^a^total does not add up to 439 as participants could choose more than one type of AMS education undertaken/received

All states and territories were represented, with approximately one-third (164/439, 37.4%) of respondents based in Victoria. Almost two-thirds were from metropolitan public hospitals (272/439, 62.0%) and one-quarter were from regional public hospitals (108/439, 24.6%). The main area of practice was hospital inpatient wards (285/439, 64.9%). Most respondents indicated that their hospital had an AMS team or individual dedicated to the review of antimicrobials (355/439, 80.9%) and most reported undertaking one or more types of AMS education (e.g. workshop or lecture) in the 12 months prior to the survey (345/439, 78.6%). A majority of respondents had postgraduate qualifications (258/439, 58.8%) and had been registered pharmacists for ≥6 years (295/434, 68.0%; 5 pharmacists did not respond to this question).

### Practice of reviewing the ongoing appropriateness of antimicrobial orders

**Fig. 1:**
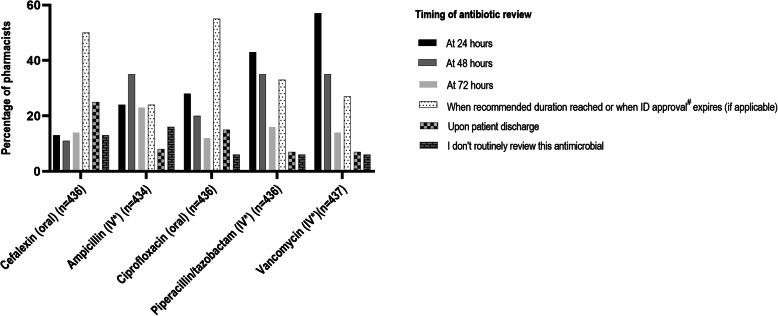
Hospital pharmacists’ practice of re-evaluating the appropriateness of antimicrobial orders after the initial prescription**.** Note that the responses for each antibiotic do not add up to 100% because participants could choose more than one time point for antibiotic review. *IV = intravenous; ^#^ID approval refers to approval for use usually obtained from the infectious diseases unit

Figure 1 shows when hospital pharmacists reported they would re-evaluate the appropriateness of different antibiotics after the initial prescription. Intravenous antibiotics (ampicillin, vancomycin and piperacillin-tazobactam), antibiotics requiring therapeutic drug monitoring (vancomycin), and broad-spectrum, typically restricted antibiotics requiring prior approval (piperacillin-tazobactam, vancomycin and ciprofloxacin) were more likely to be re-evaluated soon after prescription (within 24–72 h) compared to cefalexin (a commonly used unrestricted narrow-spectrum, oral antibiotic); most hospital pharmacists re-evaluated the latter only when the recommended duration for the infection was reached or at patient discharge (*p* < 0.01 for all comparisons between cefalexin and other antibiotics).

Seventy-nine (18.0%) respondents provided comments regarding factors affecting when they re-evaluate antimicrobial prescribing. Reasons for more frequent review included: awaiting results of microbiology tests, intravenous antibiotics (to determine switch to oral therapy), therapeutic drug monitoring requirements, patient clinical status and piperacillin-tazobactam shortage (which occurred across Australia in 2017). Working in non-inpatient areas and having a high workload led to less frequent re-evaluation. Key themes with examples of respondent quotes are available in the Additional file 1.

### Confidence in identifying AMS interventions

The level of confidence of hospital pharmacists to identify various AMS interventions upon review of antimicrobial prescribing is summarized in Table 2. Most respondents (> 65%) reported confidence in identifying a need for dose optimization based on patient-specific factors such as renal function, therapeutic drug monitoring, antibiotic allergy assessment, and discontinuing therapy when the recommended duration had been reached. Fifty percent or fewer were confident in identifying interventions related to dose optimization based on infection-specific factors such as the site of infection, bug-drug mismatch, and inappropriate lack of spectra of antimicrobial activity. In two-thirds (8 out of 12) of the survey’s clinical scenarios, a higher proportion of hospital pharmacists with ≤2 years of pharmacy registration (including intern pharmacists) reported a lack of confidence compared to more experienced pharmacists (≥3 years registration) (*p* < 0.05).

**Table 2:** Confidence of hospital pharmacists to identify antimicrobial stewardship interventions upon review of antimicrobial prescribing

Antimicrobial stewardship intervention	Confidence % (n)	≤2 years of registration % (n)	≥3 years of registration % (n)	*P* value^a^
Dose optimization based on patient-specific factors	75.0 (303/404)	67.6 (48/71)	76.6 (255/333)	0.11
Therapeutic drug monitoring	68.5 (278/406)	53.5 (38/71)	71.6 (240/335)	0.003
Antibiotic allergy assessment	67.1 (273/407)	59.2 (42/71)	68.8 (231/336)	0.19
Discontinuation, recommended duration reached	65.0 (264/406)	51.4 (36/70)	67.9 (228/336)	0.009
Streamlining according to guidelines	63.2 (258/408)	56.3 (40/71)	64.7 (218/337)	0.18
Intravenous-to-oral antimicrobial switch	63.0 (255/405)	38.0 (27/71)	68.3 (228/334)	< 0.001
De-escalating based on microbiology results	61.5 (251/408)	53.5 (38/71)	63.2 (213/337)	0.13
Inappropriate therapeutic duplication	55.2 (224/406)	39.4 (27/71)	58.8 (197/335)	0.001
Unlikely infection, antibiotics unnecessary	54.9 (224/408)	40.8 (29/71)	57.9 (195/337)	0.009
Inappropriate lack of spectra of activity	50.2 (204/406)	33.8 (24/71)	53.7 (180/335)	0.002
Bug-drug mismatch	46.8 (190/406)	32.4 (23/71)	49.9 (167/335)	0.007
Dose optimization based on infection-specific factors	44.3 (178/402)	28.2 (20/71)	47.7 (158/331)	0.003

^a^ *P* < 0.05 represents a significant difference between junior hospital pharmacists (≤2 years [including intern/pre-registration]) vs. more experienced hospital pharmacists (≥3 years)

### Knowledge in making AMS interventions

**Fig. 2:**
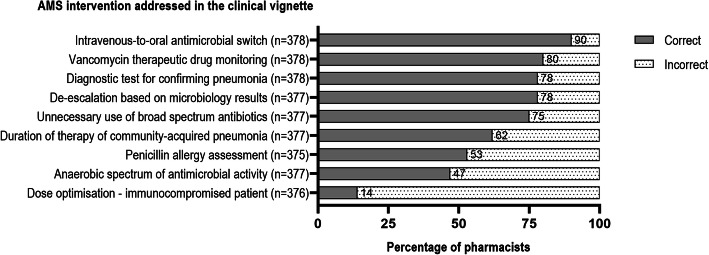
Hospital pharmacists’ knowledge in making antimicrobial stewardship interventions

The AMS interventions assessed, and the percentage of pharmacists providing a correct/incorrect answer, are shown in Fig. 2. Overall, only 13/368 (3.5%) of the respondents that completed all knowledge-based questions answered every question correctly. The median knowledge score was 6 out of 9 (IQR 5–7). Most respondents (> 80%) correctly answered questions related to intravenous-to-oral antimicrobial switch and vancomycin therapeutic drug monitoring. Approximately 50% or fewer correctly answered questions related to penicillin allergy assessment, antimicrobials’ anaerobic spectrum of activity, and recommended dose regimen of famciclovir for an immunocompromised patient.

In the linear regression analysis (Table 3), variables with a statistically significant association with relationship to knowledge score in the final multivariable model were as follows: 3–5 years of pharmacy registration, received one or more types of AMS education in the 12-months prior to the survey, and main area of hospital practice (working in AMS/ID, inpatient wards or medication safety/quality use of medicines). The overall model had an R-squared value of 0.123, indicating that the model as a whole explained 12.3% of the variance in knowledge score.

**Table 3:** Factors associated with higher antimicrobial stewardship knowledge scores for 365 participants^a^: linear regression analyses

Variable	Univariate Coefficient (95% CI)	Multivariate^b^ Coefficient (95% CI)
*State or Territory*		
Victoria	0.48 (−0.55–1.51)	–
Queensland	0.45 (− 0.64–1.53)	–
New South Wales	0.10 (−1.03–1.24)	–
Western Australia	1.19 (0.05–2.33)	#
South Australia	0.33 (− 0.81–1.48)	–
Tasmania	1.03 (− 0.06–2.11)	–
Australian Capital Territory	0.73 (− 0.87–2.33)	–
Northern Territory	(0) Reference	
*Health Service*		
Major city, public	0.51 (− 0.41–1.44)	–
Regional, public	0.15 (− 0.81–1.11)	–
Major city, private	−0.31 (− 1.61–0.98)	–
Regional, private	−0.71 (−2.19–0.77)	–
Remote or very remote public	(0) Reference	
*The hospital has an individual or team dedicated to the review of antimicrobials*		
Yes	0.28 (−0.22–0.79)	–
I don’t know	− 0.81 (− 2.20–0.57)	–
No	(0) Reference	
*Years of pharmacy registration*		
> 10 years	0.56 (− 0.37–1.49)	–
6 to 10 years	0.92 (− 0.03–1.86)	–
3 to 5 years	1.03 (0.06–2.00)	1.08 (0.12–2.04)
< = 2 years	0.37 (−0.65–1.38)	–
Intern/pre-registration	(0) Reference	
*Highest level of pharmacy education*		
Postgraduate diploma	0.55 (0.10–1.00)	#
Postgraduate certificate	0.01 (−0.53–0.55)	–
Postgraduate Master degree	0.45 (− 0.11–1.02)	–
Preregistration Master degree	−0.26 (− 1.66–1.13)	–
PhD	−1.29 (− 2.94–0.35)	–
Other (e.g. Honours)	−0.13 (− 2.12–1.87)	–
Bachelor of Pharmacy	(0) Reference	
*Current main area of hospital practice*		
Any other ward/inpatient area	1.18 (0.41–1.95)	1.11 (0.32–1.91)
Working in ward/inpatient area of high antimicrobial use (e.g. intensive care unit)	1.48 (0.62–2.34)	1.31 (0.44–2.18)
Other (e.g. manufacturing, administration, information technology)	0.66 (− 0.29–1.61)	–
Antimicrobial stewardship/infectious diseases service	2.77 (1.93–3.61)	2.19 (1.27–3.11)
Medication safety/Quality use of medicines service	1.43 (0.58–2.29)	1.26 (0.39–2.12)
Non inpatient areas (e.g. day oncology, hospital in the home [HITH])	0.57 (− 0.57–1.70)	–
Outpatient dispensary	(0) Reference	
*Antimicrobial stewardship education undertaken in the past 12 months*^c^		
	0.29 (0.17–0.41)	0.21 (0.08–0.34)

*CI* confidence interval

^a^Three-hundred and sixty-five out of the 368 participants that completed all knowledge based questions were included in the linear regression analysis because 3 participants did not answer the question regarding ‘Years of pharmacy registration’

^b^This column includes coefficient values only for variables with significant associations in the final multivariable model

^c^continuous value

#Variables with significant associations in the univariate model but were not significant in the multivariable model, and were not included in the final model

### Perceived usefulness of different modes of AMS education

**Fig. 3:**
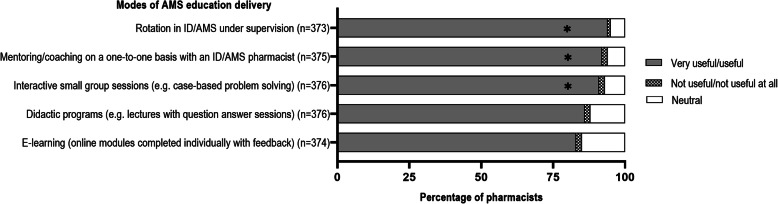
Hospital pharmacists’ perceived usefulness of different modes of education delivery relating to antimicrobial stewardship interventions *p ≤ 0.01 when comparing hospital pharmacists’ perceived usefulness of e-learning and didactic programs to other more interactive modes for responses categorized as ‘very useful/useful’. There was no difference between e-learning and didactic programs

More than 80% of respondents rated all education modes as useful or very useful, with a statistically significant difference between the more didactic modes (e-learning and lectures) and the more interactive and hands-on modes (*p* < 0.001 for all comparisons) (Fig. 3).

## Discussion

National and international guidelines recommend that all pharmacists incorporate AMS activities into their clinical practice to optimize antimicrobial prescribing and patient outcomes [6, 19]. To do this, knowledge and skills are required to confidently review the appropriateness of prescribing and identify and enact AMS interventions. To our knowledge, this is the first study to evaluate the practice, confidence and knowledge of hospital pharmacists to review antimicrobial prescribing. Our study has identified opportunities to improve hospital pharmacists’ AMS practice.

Hospital pharmacists’ practice of re-evaluating appropriateness of antimicrobial prescribing following the initial prescription review varied according to the antimicrobials presented in our survey. Compared to intravenous, broad-spectrum or restricted antimicrobials, most pharmacists indicated that they would re-evaluate the appropriateness of cefalexin (narrow spectrum, oral, unrestricted antibiotic) only when the recommended duration of therapy was reached or at patient discharge. Approximately 25% said they do not routinely review this antibiotic at all. This suggests potentially missed opportunities to avoid unnecessary or prolonged treatment. This finding may help explain the high levels of inappropriate cefalexin prescribing in Australian hospitals identified in the annual National Antimicrobial Prescribing Survey (NAPS) [20]. Results from that 2018 survey demonstrated that cefalexin is associated with a high rate of inappropriate prescribing (290/744 prescriptions, 39%). This has been a consistent finding in previous NAPS reports [21]. In the 2018 NAPS survey, other frequently prescribed, unrestricted antibiotics such as cefazolin, azithromycin, amoxicillin-clavulanic acid and metronidazole were also associated with high rates of inappropriate prescribing: 27.7 (670/2,420), 27.7 (153/552), 27.4 (352/1,286) and 25.7% (264/1,026), respectively. The most common reasons for inappropriateness included: spectrum too broad or narrow; incorrect dose, frequency, or duration; antimicrobial not required; or incorrect route [20]. The findings from our survey and the NAPS reports suggest that if hospital pharmacists were to prioritise ongoing review of these frequently prescribed antimicrobials that are not the usual focus of the AMS team, this could have a large impact on reducing inappropriate antimicrobial use.

A review of responses to the open-ended question suggests there may be barriers to hospital pharmacist review of antimicrobial use, including high workload and inadequate knowledge and confidence. An Australian study undertaken in 2014 reported barriers to optimizing antimicrobial use including insufficient pharmacy staffing [22]. They also reported some hospital pharmacists perceived antibiotic use to be a medical responsibility which they had limited capacity to influence, and a ‘low priority’ in day-to day work. Further research to identify the barriers and enablers of pharmacist involvement in AMS may be useful.

When reviewing antimicrobial prescribing, the level of confidence of hospital pharmacists to identify AMS interventions varied depending on the clinical scenario. A majority of pharmacists reported confidence to optimize doses based on patient-specific factors such renal function, therapeutic drug monitoring and allergy assessment. This is not surprising given these are common pharmacist interventions for all classes of medication. Approximately half or fewer pharmacists were confident in identifying interventions related to inappropriate spectrum of activity (inappropriate lack of spectra of activity and inappropriate double spectrum of activity), bug-drug mismatch and dose optimization based on infection-specific factors (e.g. site of infection). These are important gaps to address given the risk of therapeutic failure and subsequent impact on patient outcomes, including antimicrobial resistance; antimicrobial-resistant pathogens are associated with higher rates of mortality, illness, and prolonged hospital stay [23]. Compared to more experienced pharmacists, junior hospital pharmacists lacked confidence to identify AMS interventions. This difference highlights an opportunity for Australian undergraduate education programs to address these gaps in pharmacy curricula. It is unknown to what extent AMS education is integrated in undergraduate pharmacy curricula in Australia and how well student pharmacists are prepared to apply AMS strategies in clinical practice. A cross-sectional, multicentre survey of 116 faculty members from various colleges and schools of pharmacy in the United States revealed there was variability in the extent, content and methodology of AMS education in Doctor of Pharmacy curricula [24].

The median AMS knowledge score in our study was reasonably good (6 out of 9). This may be because more than half of the respondents had ≥6 years of practice experience (68.0%), a post-graduation qualification in pharmacy (58.8%), and received education in AMS in the 12 months prior to the survey (78.6%). Respondents’ perceived lack of confidence in identifying AMS interventions related to antimicrobial spectra was also reflected in the knowledge test where only 47% pharmacists provided a correct answer to a question on this topic. Interestingly, respondents in three North American studies that included a similar knowledge question performed better. Fifty-three percent of 317 medical students across three universities and 60% of 578 Doctor of Pharmacy students from 12 schools of pharmacy provided a correct answer [8, 12]. These higher scores may reflect different education and training received on antimicrobial pharmacology and AMS at an undergraduate level or other factors such as timing of the surveys in relation to receiving AMS education. Two-thirds of 402 prescribing clinicians (physicians, physician-assistants and nurse practitioners) at five hospitals also provided a correct answer [16]. A published teaching tool on spectrum of antibiotic activity is available and can be adopted by hospital pharmacists to improve their knowledge in this area [25].

Another identified knowledge gap was the appropriate antibiotic choice in a patient with a reported penicillin allergy which is a known non-allergy side effect. This occurred despite two-thirds of hospital pharmacists reporting confidence in identifying a patient with a true antimicrobial allergy. Since the completion of this study, resources that include an assessment tool to better clarify the nature of penicillin allergies and improve prescribing, have been developed to upskill clinicians, including pharmacists [26, 27]. Only 13% of hospital pharmacists provided a correct answer regarding the dose regimen for famciclovir in an immunocompromised patient, although most respondents selected ‘I’m not sure. I would have to look it up’ suggesting, this question was too difficult for respondents to answer unaided.

When controlling for other factors, more experienced pharmacists (3–5 years of pharmacy registration) performed better in the knowledge test compared to interns and newly registered pharmacists (<=2 years of registration). This suggests undergraduate and pre-registration education could be enhanced so that intern and newly registered pharmacists are job-ready, rather than requiring around 3 years to develop good knowledge and confidence in AMS. Working in an inpatient area (AMS/ID, inpatient wards or medication safety/quality use of medicines) was another predictor of higher knowledge scores, possibly due to increased opportunities to review antimicrobial prescribing and associated clinical and laboratory data compared to working in non inpatient areas (e.g outpatient dispensary). Whilst undertaking an AMS education activity (e.g. lectures, workshops etc.) in the 12 months prior to the survey was also a predictor for higher knowledge scores, it appeared that it had only a limited effect. This suggests that more effective modes of education delivery may be needed to provide the knowledge and confidence necessary to identify and enact AMS interventions. The variables included in the final multivariate model explained only about one-eighth of the variance in knowledge score, suggesting other factors (e.g. workload, attitude, workplace culture or quality of AMS education undertaken pre or post registration) that were not measured contributed to the variance.

When asked about the usefulness of various modes of AMS education delivery, a rotation in ID/AMS under supervision, one-on-one mentoring/coaching and interactive small group tutorials were perceived to be useful or very useful by a higher proportion of respondents than more didactic options such as lectures and e-learning modules. However respondents were asked whether each type of education would be useful, rather than being asked to rank their relative usefulness. The latter approach may have provided better insights into pharmacists’ preferences for education delivery.

There is limited research evaluating the impact of educational interventions or comparing different interventions to improve hospital pharmacists’ knowledge and skills in AMS. One study conducted in the United States described the development and evaluation of an educational and training program targeted at pharmacists in a community hospital without an ID/AMS pharmacist on site [28]. Multiple didactic and active educational strategies (lectures, small group sessions and online learning) were employed to teach AMS interventions such as intravenous-to-oral switch, and dose-adjustment based on renal function. Pharmacists’ knowledge was assessed pre- and post the program using primarily short-answer questions involving various scenarios related to AMS. The mean pre- and post-education test scores were 49.7% vs 79.2%, (*p* < .001) indicating a substantial improvement in AMS knowledge. A South African study employing a novel AMS comic book to teach hospital pharmacists general ID principles and AMS concepts showed a significant improvement in knowledge based on pre- and post-test scores (66% vs. 96%) (*p* < 0.05) [29]. None of the aforementioned studies evaluated the impact of their interventions on appropriateness of antimicrobial use. However, an Australian study across 14 hospitals compared the effect of a vancomycin TDM interactive e-learning tool to a standard didactic email intervention on nurses, pharmacists and doctors knowledge and clinical use of vancomycin (proportion of plasma trough levels falling within the recommended range) [30]. Compared to nurses and doctors, pharmacists’ mean knowledge scores were high overall pre- and 4-months post-interventions although there were no significant post-intervention differences in the proportion of vancomycin trough levels within the recommended range.

Our study has a number of strengths and limitations. Strengths include a relatively large sample size including good representation of hospital pharmacists working in regional hospitals as well as metropolitan hospitals. Limitations include a low response rate, however, our sample size of 439 is greater than the recommended minimum sample size of 346 for a survey of a population of 3500 people [31]. It should also be noted that we surveyed members of SHPA and not all hospital pharmacists recorded in the Australian health practitioner regulation agency (*N* = 5451 in 2017). There is a risk of response bias given the survey was voluntary, which might have led to an over-estimate of levels of confidence and median knowledge score because those who lacked confidence or knowledge may not have answered. Furthermore, some respondents might have used external resources to answer the questions, leading to an over-estimate of their knowledge. However, were this to be the case the identified gaps in practice, knowledge and confidence are likely to be even greater. Also, we did not test respondents’ confidence and knowledge on all AMS interventions because to do this would have negatively affected the length of the survey. Lastly, this study was conducted in Australia therefore, differences in AMS education and training at an undergraduate and postgraduate level between countries, might affect generalisability of the results.

## Conclusion

This survey identified gaps in the practice, confidence and knowledge of hospital pharmacists in relation to reviewing antimicrobial prescribing and undertaking AMS interventions. It highlights areas to enhance and integrate AMS in hospital pharmacists’ clinical practice to improve antimicrobial prescribing and patient outcomes. Our findings can inform the development of education and training programs for pharmacists. Further research to evaluate the impact of such programs on hospital pharmacists’ knowledge, skills, and AMS practice is warranted.

## Supplementary Information

**Additional file 1.** Survey & key concepts with examples of respondent comments regarding factors affecting when they re-evaulated antimicrobial prescribing. Survey used in the study to collect data. Key concepts with examples of respondent comments regarding factors affecting when they re-evaulated antimicrobial prescribing. (Optional free-text comments associated with question 8 in the survey).

## Data Availability

The datasets generated and/or analysed during the current study are not publicly available due to participant privacy but are available from the corresponding author on reasonable request.

## Abbreviations

AMS: Antimicrobial stewardship
ID: Infectious diseases
PAF: Prospective audit and feedback
MCQ: Multiple choice questions
SHPA: Society of Hospital Pharmacists of Australia
NAPS: National Antimicrobial Prescribing Survey
