# Supplementary material for: Exploring the practice, confidence and educational needs of hospital pharmacists in reviewing antimicrobial prescribing: a cross-sectional, nationwide survey
Source: BMC Med Educ. 2021 Apr 23;21:235. doi: 10.1186/s12909-021-02664-1 (PMC8066433; doi:10.1186/s12909-021-02664-1)
Supplement: Supplementary file 1 — Additional file 1. Survey & key concepts with examples of respondent comments regarding factors affecting when they re-evaulated antimicrobial prescribing. Survey used in the study to collect data. Key concepts with examples of respondent comments regarding factors affecting when they re-evaulated antimicrobial prescribing. (Optional free-text comments associated with question 8 in the survey). [file 12909_2021_2664_MOESM1_ESM.docx]

**Additional file**

**Exploring the practice, confidence and educational needs of hospital pharmacists in reviewing antimicrobial prescribing: a cross-sectional, nationwide survey.**

1. **Survey**

**Demographic information**


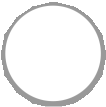

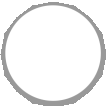

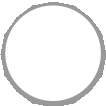

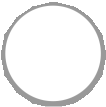

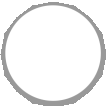

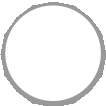

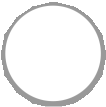

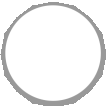

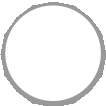

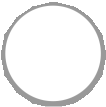

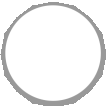

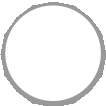

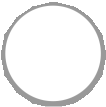

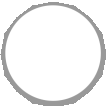

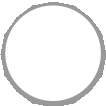

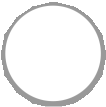

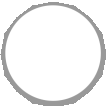


* 1. Which State/Territory do you work in?

Australian Capital Territory New South Wales Northern Territory Queensland

South Australia Tasmania Victoria

Western Australia

- 2. How would you best describe the health service you primarily work at?

Major city, public Major city, private Regional, public Regional, private

Remote or very remote, public Remote or very remote, private

#### Does your hospital have an individual or team dedicated to the review of antimicrobials after they are prescribed?

Yes No

I don't know

#### What is your current main area of hospital practice?


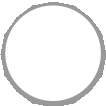
 Working in ward/inpatient areas of high antimicrobial use including ICU, transplant wards, haematology/oncology, ID or burns units


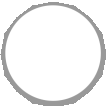
 Any other ward/inpatient area
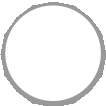
 Outpatient dispensary


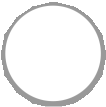
 Ambulatory care (HITH, Day oncology)


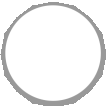
 Medication safety / Quality Use of Medicines
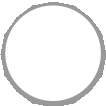
 Antimicrobial stewardship or Infectious Diseases

Other (please specify)

#### How many years have you been registered as a pharmacist?


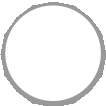
 I am an intern/pre-registration pharmacist
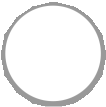
 <= 2 years


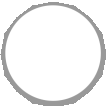
 3 to 5 years


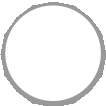
 6 to 10 years


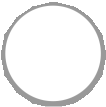
 >10 years

#### What is your highest level of pharmacy education?


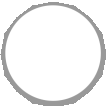
 BPharm


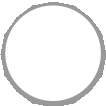
 Preregistration Master
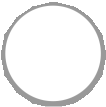
 Postgraduate Certificate
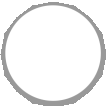
 Postgraduate Diploma
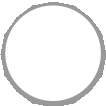
 Postgraduate Master
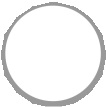
 PhD

Other (please specify)

#### Please specify the type of education or training in infectious diseases (ID) and/or antimicrobial stewardship (AMS) you have undertaken/received in the **past 12 months**. (Tick all that apply.)

I have not received/undertaken any education or training in ID/AMS Postgraduate studies (university)

Workshop or seminar Lecture

Web-based module/course

Informal education in the clinical workplace Self-directed learning

Other (please specify)

## Your practice regarding the review of an antimicrobial after it is prescribed

#### After initial review of an antimicrobial order for an inpatient, when would you re-evaluate the appropriateness* of the antimicrobials listed below. (Tick all that apply.)

*appropriateness = right antimicrobial choice, dosage, route and duration

Cefalexin (oral) Ampicillin (IV) Ciprofloxacin (oral)

Piperacillin+tazobactam

(IV)

Vancomycin (IV)

At 24 hours At 48 hours At 72 hours

When recommended duration

reached or when ID approval expires (if applicable)

Upon patient discharge

I don't routinely review this antimicrobial

Please add comments if necessary

## Your perception regarding AMS interventions upon review of an antimicrobial after it is prescribed.

#### How confident do you feel identifying the following scenarios?


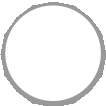

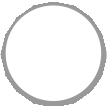

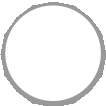

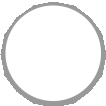

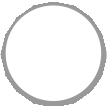

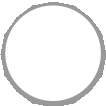

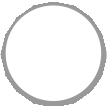

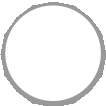

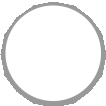

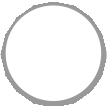

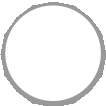

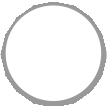

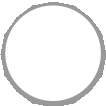

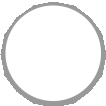

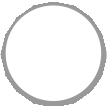

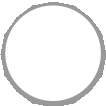

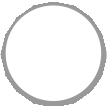

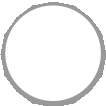

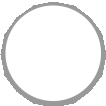

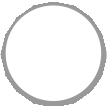

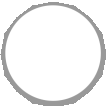

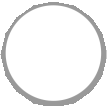

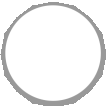

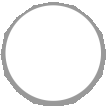

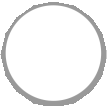

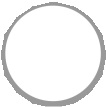

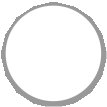

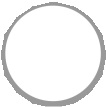

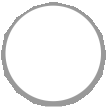

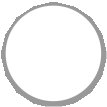


**Unlikely infection** Identifying a patient receiving antimicrobial(s) without any positive culture data or confirmatory diagnostic testing

**Discontinuation of antimicrobials** Identifying antimicrobial order(s) that are no longer necessary after the appropriate duration of

therapy has been reached

##### Streamlining

Identifying antimicrobial therapy which differs from national or local guidelines

**De-escalating** Identifying where therapy could be changed from a broad-spectrum antimicrobial to a narrow- spectrum antimicrobial based on

culture/susceptibility results

##### IV to oral switch

Identifying a patient on

IV antimicrobials who has improved clinically, is able to tolerate oral medications and a suitable oral alternative is available

##### Drug dosing/optimisation 1

Identifying antimicrobial orders that differ from recommended doses based on patient-specific factors such as weight, creatinine clearance or immune status.

Not confident at all

Somewhat

confident Confident Very confident

I don't know how I feel


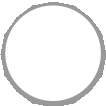

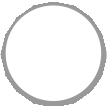

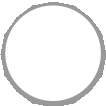

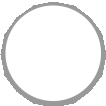

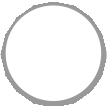

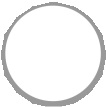

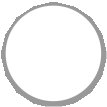

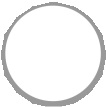

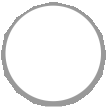

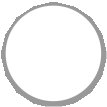

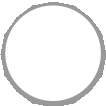

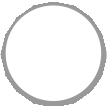

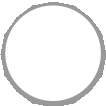

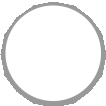

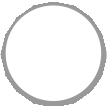

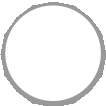

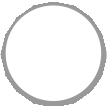

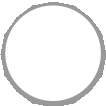

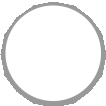

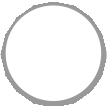

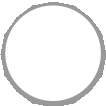

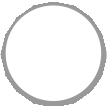

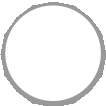

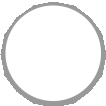

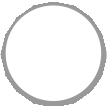

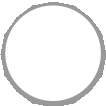

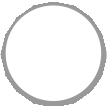

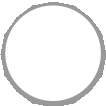

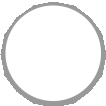

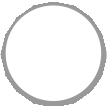


##### Drug dosing/optimisation 2

Identifying antimicrobial orders that differ from recommended doses based on infection-specific factors such as site of infection and organism.

**Antibiotic allergies** Identifying a patient with a true antimicrobial allergy after conducting a thorough assessment of the nature of the allergic reaction i.e. specific drug, timing of onset, other antimicrobial exposure since reaction etc.

**Therapeutic Drug Monitoring (TDM)** Recommending antimicrobial TDM and interpreting results

##### Inappropriate double coverage

Identifying patients on duplicative antimicrobial spectra of activity (e.g. piperacillin+tazobactam and metronidazole)

**Inappropriate lack of spectra of activity** Identifying patients where the antimicrobial regimen is missing spectra of activity for the infection being treated (e.g. missing doxycycline for moderate community-acquired pneumonia)

**Bug-drug mismatch** Identifying where an organism is likely to be resistant to a patient’s current antimicrobial regimen

Not confident at all

Somewhat

confident Confident Very confident

I don't know how I feel

## Your knowledge in making these AMS interventions


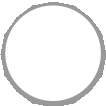

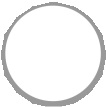

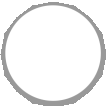

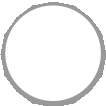

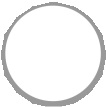

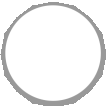


**Please answer the following clinical vignettes regarding AMS interventions providing a SINGLE best possible answer. We understand that it is tempting to look up answers, but please do not use references and answer honestly based on your current knowledge. It is OK if you don’t know the answer as we are looking at ways to improve this.**

- 1. Steven Jones was admitted to your ward post coronary artery bypass surgery. The next day he was prescribed ceftriaxone for possible hospital acquired pneumonia as he complained of a cough and had an elevated C-reactive protein. Which of the following sources of information would best confirm the diagnosis of pneumonia?

Sputum cultures Serum creatinine Chest x-ray White blood cells Temperature

I am not sure. I would have to look it up

#### What is the recommended duration of treatment (IV + oral) for community-acquired pneumonia of moderate severity in an adult?

10 days

7 days

5 days

3 days

I am not sure. I would have to look it up

#### Piperacillin + tazobactam is NOT routinely indicated in which of the following clinical scenarios?

Diabetic foot infections Cellulitis

Biliary sepsis

Hospital acquired pneumonia Febrile neutropenia

I am not sure. I would have to look it up

#### You review a blood culture positive for*Escherichia coli* susceptible to ampicillin, piperacillin+tazobactam, gentamicin and ciprofloxacin in a 90-year old patient with urosepsis who was initially treated with ceftriaxone 1g, IV, daily. He has no allergies. What recommendation would you make to the treating team with respect to **intravenous** antibiotic treatment?

Continue current treatment Switch to ciprofloxacin

Switch to ampicillin

Switch to piperacillin + tazobactam Switch to gentamicin

I am not sure. I would have to look it up

#### Joan Smith is hospitalised with fevers, chills and productive cough. A diagnosis of community acquired pneumonia is made and she is prescribed intravenous benzylpenicillin and oral doxycycline. After 48 hours she shows clinical improvement; afebrile, feels better and eating but still has a productive cough. Blood cultures are negative. What recommendation would you make to the treating team?

Continue current therapy Add oseltamivir

Switch to ceftriaxone

Switch to oral amoxicillin (plus oral doxycycline) Cease antimicrobial therapy

I am not sure. I would have to look it up

#### An immunocompromised patient, without known renal impairment, is treated with famciclovir 250mg orally, 8-hourly for 7 days for herpes zoster. What recommendation, if any, would you make?

No change; dose regimen is appropriate I’d recommend an increase in the dose I’d recommend a increase in the duration

I’d recommend an increase in the dose and duration I am not sure. I would have to look it up

#### Vancomycin (1.5g IV 12-hourly) is being used for a methicillin-resistant*Staphylococcus aureus* (MRSA) endocarditis in a patient with normal renal function. A trough (predose) level, taken before the fourth dose, is 11 mg/L. What would you do about the vancomycin dose?

I wouldn’t change the dose because it is therapeutic I’d increase the dose

I’d increase the frequency of dosing I’d decrease the dose

I'd decrease the frequency of dosing I am not sure. I would have to look it up

#### Which of the following antimicrobials does NOT cover anaerobes?

Metronidazole

Piperacillin+tazobactam Clindamycin

Amoxycillin+clavulanic acid Ciprofloxacin

I am not sure. I would have to look it up

#### Mrs J Smith is diagnosed with methicillin-sensitive *Staphylococcus aureus* (MSSA) bacteraemia and prescribed vancomycin because of a history of penicillin allergy. She describes the allergy as severe vomiting which occurred after taking Augmentin Duo Forte ® for a urinary tract infection 5 years ago (her local doctor switched her antibiotic but gave no other treatment). What do you recommend?

Continue vancomycin and check pre dose/trough level prior to the 4th dose Change to cefazolin

Change to IV flucloxacillin Change to meropenem

I am not sure. I would have to look it up

#### Please rate your preference with regard to each of the following options for learning about pharmacist- led AMS interventions in the workplace.

Very useful Useful Neutral Not useful Not at all useful

Didactic programs (e.g. lectures with question answer sessions)

Interactive small group sessions (e.g. case- based problem solving)

e-Learning (e.g. online modules completed individually with feedback)

Mentoring/coaching on a one-to-one basis with a pharmacist knowledgeable in ID/AMS

A rotation in ID or AMS under supervision

Other (please specify)

# Thank you for your time and participation

1. **Key concepts with examples of respondent comments regarding factors affecting when they re-evaluated antimicrobial prescribing. (Optional free-text comments associated with Question 8 in the survey.)**

| **Availability of results of microbiology tests**  *“Would also depend in micro results available, including sensitivities - I will often flag when these results available for follow up” (Pharmacist 337)*  *“Also ideally check* [antimicrobial prescribing] *when cultures come back for sensitivities” (Pharmacist 102)*  *“This would depend on when MCS results were available. I would review every 24hours until sensitivities were known, and then my practice would change.” (Pharmacist 128)*  *“If cultures come back positive with antibiotic sensitivities after 24-48hrs then would review choice of antibiotic then” (Pharmacist 68)* |
| --- |
| **Intravenous antibiotics**  *“Generally IV antimicrobials reviewed on daily basis as renal function and cultures may be back requiring changes or modification to therapy” (Pharmacist 209)*  *“IV antibiotics reviewed daily. Oral antibiotics reviewed when suggested course complete” (Pharmacist 394)*  *“I would be reviewing the oral when recommended duration is reach, and IV at 48 hours” (Pharmacist no 190)* |
| **Therapeutic drug monitoring requirements**  *“Vancomycin is reviewed based on TDM which requires trough levels taken usually immediately before the third or fourth dose depending on whether a loading dose was given” (Pharmacist 45)*  *“Vancomycin would depend on the timing of the first level” (Pharmacist 197)*  *“For vancomycin it depends on when levels are taken”* *(Pharmacist 374)* |
| **Clinical status of the patient**  *“usually review antimicrobials daily for any acute changes in patient” (Pharmacist 74)*  *“You would also take into account the clinical condition (particularly for intravenous therapy) (Pharmacist 386)*  *“I would review antibiotics if blood test change e.g. if LFT or Cr goes off…” (Pharmacist 281)* |
| **Piperacillin-tazobactam shortage**  *“Due to Tazocin shortage, I have been re-evaluating the use of this antibiotic after a shorter time interval than I previously used to.” (Pharmacist 282)*  *“About to implement PipTaz restrictions due to shortage, and will require screening of all orders during working hours…” (Pharmacist 254)* |
| **Area of hospital pharmacy practice**  *“I work in ED where ongoing therapy review does not usually happen as patients have moved on” (Pharmacist 288)*  *“Don't work in inpatients” (Pharmacist 274)*  *“Work in palliative care - don't see IV usage, and often oral has other factors at play.” (Pharmacist 41)* |
| ***High workload***  *“High workload making it hard to keep up the review” (Pharmacist 279)*  *“I am unable to review all patients. Therefore the majority I wouldn’t see until discharge” (Pharmacist 368)*  *“Sole Pharmacist on 40 bed medical ward (110% occupancy) in area with high demographics of elderly patients and low social economic- Opportunities to review are limited” (Pharmacist 302)* |
